# Electrochemical and Spectrophotometric Methods for Polyphenol and Ascorbic Acid Determination in Fruit and Vegetable Extracts

**DOI:** 10.17113/ftb.58.02.20.6593

**Published:** 2020-06

**Authors:** Fiorella Tulli, María Laura Lemos, Diego R. Gutiérrez, Silvia del C. Rodríguez, Beatriz A. López de Mishima, Verónica I. Paz Zanini

**Affiliations:** 1 NOA Institute of Bionanotechnology (INBIONATEC), CONICET, National University of Santiago del Estero (UNSE), RN9, Km 1125, G4206XCP Santiago del Estero, Argentina; 2 Research Center in Applied Biophysics and Food (CIBAAL), CONICET, National University of Santiago del Estero (UNSE), RN 9, Km 1125, G4206XCP Santiago del Estero, Argentina; 3 Institute of Food Science and Technology (ICyTA), Faculty of Agronomy and Agroindustry National University of Santiago del Estero-Argentina, Av. Belgrano (S) 1912, G4206XCP Santiago del Estero, Argentina.

**Keywords:** electrochemical and spectrophotometric method comparison, green leafy vegetables, eggplant, carrots, ascorbic acid and polyphenols

## Abstract

**Research background:**

Fresh-cut fruits and vegetables are considered sources of antioxidant compounds. However, their shelf life is limited due to nutritional, quality and safety deterioration. Therefore, in recent decades, various methods have been reported for food processing and preservation, as well as for the determination of antioxidant compounds, due to their many benefits when consumed. The aim of the present work is to compare the performance of electrochemical and spectrophotometric methods in the analysis of the content of polyphenolic compounds and ascorbic acid in extracts from fruits (eggplant), edible roots (carrot) and leaves (rocket, lettuce and chard), and evaluate their capability to detect small changes in the antioxidant content in the eggplant extracts previously irradiated with different UV-C light intensities.

**Experimental approach:**

Polyphenolic compounds and ascorbic acid were determined by electrochemical and spectrophotometric methods. An enzymatic biosensor and a nanocomposite sensor were used for polyphenolic compounds and ascorbic acid, respectively, in electrochemical measurements, while Folin-Ciocalteu and Kampfenkel methods were used for spectrophotometric measurements.

**Results and conclusion:**

Results obtained through the different methodologies were comparable and consistent with each other. Both methods allowed determining the content of ascorbic acid and polyphenolic compounds in the fruit and vegetable extracts. Moreover, both techniques enable the detection of the analyte concentration changes in samples exposed to different UV-C intensities and storage days. Finally, it was observed that the antioxidant capacity depends on the type of food, treatment and storage period.

**Novelty and scientific contribution:**

Both methodologies were suitable for the quantification of analytes; however, the electrochemical sensors provided higher specificity and selectivity, applicable to different fruit and vegetable matrices, obtaining results with higher precision, in shorter time and with a smaller sample volume, minimizing the economic costs because of the lower consumption of reagents.

## INTRODUCTION

Fruits and vegetables are the main natural sources of the most important antioxidants in diet, such as polyphenolic compounds (PPhC) and ascorbic acid (AA). These compounds have been reported to be potentially applicable for the prevention of some chronic and neurodegenerative diseases (*1*, *2*). Root vegetables such as carrots (*Daucus carota*) and green leafy vegetables such as rocket (*Eruca sativa* Mill.), lettuce (*Lactuca sativa*) and chard (*Beta vulgaris*) are examples of highly nutritious foods due to their high antioxidant content (β-carotene, polyphenols, ascorbic acid, chlorogenic acid, *etc*.), vitamins (A, C, complex B, E and K), minerals (calcium, iron, magnesium, potassium) and fibre. In particular, eggplant (*Solanum melongena* L.) is one of the fruits with the highest antioxidant capacity due to the high content of bioactive substances.

In the last decades, the consumption of safer and healthier fruits and vegetables has increased because consumers seek to improve the therapeutic and nutritive quality of their diet (*3*). A widely used method in food processing is to offer fresh-cut vegetables and fruits that maintain their *in natura* state, without altering their intrinsic characteristics. This method allows consumers to perceive food attributes, such as fresh appearance and flavour, and also make them easier to consume. However, fresh-cut vegetable and fruit products are more sensitive to deterioration than the non-processed ones. In particular, mechanical injury promotes microbial growth and biochemical changes, producing a decrease in product quality and safety (*4*). Therefore, the development and application of technologies aimed at preserving the quality of fresh-cut products are required.

Non-thermal treatments are particularly interesting because they allow the preservation of the nutritional and physicochemical food properties, and are more energy-efficient processes (*5*). Among all these treatments, the most studied in recent years have been high hydrostatic pressure (HHP), pulsed electric fields (PEF), ultrasound (US), ultraviolet light (UV), and cold plasma (CP). Each technique has specific microbial inactivation mechanisms: in HHP microbial death is caused by the changes in cell membrane (*6*), and in PEF by electrochemical compression and osmotic imbalance (*7*). Cavitation is the basic mechanism that causes microorganism destruction in the US (*8*), while in the CP the intracellular accumulation of charged particles may induce apoptosis, electrostatic disruption and electroporation (*9*). Microbial inactivation mechanism by UV-C is mainly based on nucleic acid alteration and cytoplasmic membrane damage (*10*). The method to be used will depend on the processing parameters, microbial strain and nutritional properties.

UV-C radiation is one of the easy-to-use physical techniques with proven effectiveness to control deterioration of different fruits and vegetables. Although its effect is only on the cutting surface, it is highly effective in extending the shelf life of fresh-cut products, where microbiological and enzymatic damage mainly occurs (*4*). The inhibition of microbial growth is due to pyrimidine dimer formation that alters the DNA helix and blocks microbial cell replication. Therefore, cell death occurs because they cannot repair DNA damaged by radiation. The effective dose of UV-C depends on the type of the irradiated vegetable, and too high doses can cause harmful effects on the product quality (*11*). UV-C light treatments are an excellent alternative, since they do not leave residues, are less expensive, lethal to many microorganisms and have no legal restrictions. It has been reported that UV-C light can also activate secondary metabolism and, consequently, induce the synthesis of antioxidant compounds (*4*).

Several methods have been reported to evaluate the total PPhC and AA content and antioxidant capacity in fruits, vegetables, fruit drinks, beverages, powdered drinks, *etc*. For the qualitative and quantitative evaluation, Raman (*12*, *13*), Fourier transformed infrared spectroscopy (FTIR) (*14*) and high-performance liquid chromatography (HPLC) (*15*, *16*) are used. However, for practical approaches, a faster and easier determination of PPhC and AA is needed. For this purpose, spectrophotometric assays such as ferric reducing antioxidant power (FRAP) (*17*), cupric reducing antioxidant power (CUPRAC) (*18*), total radical-trapping antioxidant parameter (TRAP) (*19*), 2,2-diphenyl-1-picrylhydrazyl (DPPH) (*20*), 2,2′-azinobis-(3-ethylbenzthiazoline-6-sulfonate) (ABTS) (*21*), Folin-Ciocalteu (*22*), and Kampfenkel are widely applied (*23*).

Although good analytical results are obtained, most of them require laborious preliminary treatment steps, expensive equipment and reagents, and are not ecofriendly. In this sense, electrochemical sensors are presented as an excellent alternative for the control of composition and quality, and the monitoring of all stages in food production process due to several advantages such as fast response, specificity, reliability, less expensive equipment, less laborious sample preparation and analysis conditioning, and analysis *in situ*.

Nevertheless, the direct electrochemical determination of PPhC and AA is complicated due to electrode fouling as a result of product surface adsorption or the low diffusion and electron transfer rate (*24*-*26*). Fortunately, advances in nanotechnology empowered the design and development of various materials such as metallic nanoparticles (*27*), quantum dots (*28*), carbon-based materials (graphene, carbon nanotubes, carbon black, *etc*.) (*29*), semiconductors, among others, that improved the performance of electrochemical sensors (*29*, *30*), as well as the spectrophotometric ones (*27*), allowing the PPhC and AA determination (*31*, *32*).

Therefore, this work aims to compare the electrochemical and spectrophotometric methods for PPhC and AA determination in extracts from roots, green leafy vegetables and fruits. Besides, we evaluated the capability of electrochemichal sensors to detect concentration changes of PPhC and AA in fresh-cut eggplants exposed to different UV-C light intensities during the storage period.

## MATERIALS AND METHODS

### Materials

Commercial synthetic hectorite Laponite RD (monovalent cation exchange capacity CEC=0.74 mmol/g) was obtained from Laportes Industries (Luton, UK). Horseradish peroxidase lyophilized powder (EC 1.11.1.7, 193 U/mg), citrate buffer-stabilized spherical gold nanoparticles (particle size (5±1) nm), hydroquinone, caffeine, chlorogenic, ascorbic, citric, glutamic, tartaric acids and tetrachloroauric(III) acid trihydrate, 20% (*m*/*V*) in water polydiallyldimethylammonium chloride, sodium hydroxide, sodium carbonate, and Folin-Ciocalteu reagent were purchased from Sigma-Aldrich S.A, Merck (St. Louis, MO, USA). Multiwall carbon nanotubes (outer diameter (30±15) nm, length 1-5 μm) were purchased from NanoLab Inc (Waltham, MA, USA). Sulfuric, nitric and tetrachloride acetic acids, 2,2'-dipyridyl and iron(III) chloride were from Biopack (Buenos Aires, Argentina). Phosphoric acid and absolute ethanol were obtained from Dorwill (Buenos Aires, Argentina). Reagent grade buffer phosphate sodium salts NaH_2_PO_4_ and Na_2_HPO_4_, hydrogen peroxide (30%) and glucose were obtained from J.T.Baker (Phillipsburg, NJ, USA). Water-soluble polystyrene copolymers of vinylbenzylthymine (VBT) and vinylbenzyl triethylammonium chloride (VBA) ({[(VBT)(VBA)_4_]^4+^}_25_) or VBT and vinylphenyl sulphonate (VPS), ({[(VBT)(VPS)_16_]^16-^}_25_) were synthesized as described before (*33*, *34*). In all cases, the solutions were prepared using triple distilled water.

### Extract preparation

Eggplants (*Solanum melongena* L. cv. Black Nite), carrots (*Daucus carota* L. cv. Chantenay) and green leafy vegetables rocket (*Eruca Sativa* Mill.), lettuce (*Lactuca sativ*a cv. *Capitata*) and chard (*Beta vulgaris* cv. Cicla) were purchased from a local producer (Santiago del Estero, Argentina). After harvest, the products were stored at (4±1) °C (Briket Master 5000; Buenos Aires, Argentina) until used. Before extract preparation, vegetables were washed in chlorate water and air dried.

The PPhC determination by electrochemical and spectrophotometric methods was carried out with extracts obtained by homogenizing fresh eggplants and carrots (1 and 5 g, respectively) with 20 mL of methanol.

The extracts for AA electrochemical determination were prepared using 8 g of fresh eggplants and 12 mL of 0.1 M phosphate buffer solution, pH=2, and 5 g of green leafy vegetables and 10 mL of the same buffer solution. For spectrophotometric determination, eggplant and green leafy vegetables were prepared following the same procedure but using 6% (*m*/*V*) trichloroacetic acid (TCA) as solvent.

In all cases, after homogenizing the vegetable tissues with the solvent, samples were filtered (Whatman^®^ grade 589/2, *Ф*=100 mm) and centrifuged at 10 000×*g* for 15 min at 4 °C (Ependorf 5430; Thermo Fisher, Madrid, Spain).

### Eggplants exposed to UV-C light treatments

Fresh-cut eggplants were exposed to different UV-C light intensities for 300 s, using a home-made reflective stainless steel chamber (1.70 m×1.00 m×0.10 m) containing 12 fluorescent germicidal lamps (254.7 nm, TUV 36W/G36; Philips, Amsterdam, The Netherlands) distributed equally in the upper and lower part of the steel chamber (*35*). Samples were divided into three groups according to the UV-C light intensity treatment: (*i*) untreated used as control, (*ii*) 1.0, and (*iii*) 10.0 kJ/m^2^. Radiation intensity was monitored with a portable digital radiometer (Cole-Parmer Instrument Company, Vernon Hills, IL, USA). Samples were packed in polypropylene (PP) trays (17.4 cm×13.8 cm×4.8 cm; Cellpack SA, Santa Fe, Argentina), heat-sealed with 35 μm thick PP film (O_2_ permeability: 2.58·10^-6^ mol/(m^2^·s·Pa) and CO_2_ permeability: 9.30·10^-6^ mol/(m^2^·s·Pa) at 25 °C and 90% RH (data provided by National Institute of Industrial Technology, Santiago del Estero, Argentina), and stored at (4±1) °C in the fridge (Briket Master 5000). The vegetable extracts were prepared as described in *Extract preparation*, and the changes in the PPhC and AA contents were tested by electrochemical and spectrophotometric assays on days 0, 2, 4 and 6 of storage, considering that fresh-cut fruits and vegetables are available in the market for a maximum of 6-7 days.

### Electrochemical method

Measurements were carried out at room temperature in a three-electrode electrochemical cell with 0.1 M phosphate buffer as supporting electrolyte. A platinum wire, an Ag|AgCl|Cl^-^ (3 M) and different modified glassy carbon electrodes (GCE; CH Instruments, Bee Cave, TX, USA) were used as counter, reference, and working electrodes, respectively. Amperometry was performed with a potentiostat/galvanostat (model Teq4; nanoTeq, Buenos Aires, Argentina) under convective conditions, and the working potential was chosen in order to reach stationary state conditions.

For PPhC determination, the GCE surface was modified with a hydrogel composed of Laponite RD, citrate buffer-stabilized spherical gold nanoparticles, copolymers of vinylbenzylthymine (VBT) and vinylbenzyl triethylammonium chloride (VBA) ({[(VBT)(VBA)_4_]^4+^}_25_), and horseradish peroxidase enzyme (HRP), as described in our previous work (*31*). PPhC determination is based on the cyclic enzymatic reaction of HRP. The enzyme is oxidized by the action of hydrogen peroxide. The oxidized enzyme takes electrons from the PPhC, which are ultimately reduced at the electrode surface at -200 mV (working potential), resulting in a reduction current proportional to the PPhC concentration. The high specificity towards PPhC and catalytic nature of the immobilized HRP allows obtaining an amplified electrical signal proportional to analyte concentration, without the interference of ascorbic, tartaric or citric acids (*31*), potential interferences in food analysis.

On the other hand, functionalized multiwall carbon nanotubes (fMWCNT), poly(diallyldimethylammonium chloride) capped gold nanoparticles (AuNP@PDDA, particle size (12±2) nm), and copolymers of vinylbenzylthymine (VBT) and vinylphenylsulphonate (VPS) ({[(VBT)(VPS)_16_]^16-^}_25_) were used to modify the GCE surface. A working potential of 50 mV was applied to determine the AA mass fraction. Although in this case a biorecognition element was not immobilized on the working electrode, the inclusion of nanomaterials favours complete oxidation of AA at a low positive potential with a high sensitivity (*32*).

Mass fractions of both analytes were determined by the standard addition method using the extract supernatant. Before the sample addition, two successive aliquots of a standard solution were aggregated to verify proportionality between the current signal and the concentration (*31*, *32*). To evaluate the possible food matrix effects, the apparent recovery (R_A_) of the signal produced by a third aliquot of the standard solution was measured (*36*). The standard solutions used to carry out the determination of PPhC and AA were chlorogenic acid (ChlA) and AA, respectively.

Spectrophotometric method

A calibration curve was performed according to Singleton and Rossi (*22*). Different volumes of ChlA standard solution were mixed with 50 μL Folin-Ciocalteu reagent (1 M). Next, 100 μL Na_2_CO_3_ (20%, m/*V*) in 0.1 M NaOH were added, and after sonication in darkness at 25 °C for 60 min, the absorbance was measured at 765 nm in a UV-Vis spectrophotometer (model 8453; Agilent Technologies, Santa Clara, CA, USA). The PPhC concentration was determined according to the sample absorbance and the Lambert-Beer law.

The procedure described by Kampfenkel (*23*) was used to carry out the calibration plot for AA determination. Different concentrations of an AA standard solution were mixed with 0.2 M phosphate buffer at pH=7, 1 mL of 10% (*m*/*V*) TCA, 800 μL of 42% (*V*/*V*) H_3_PO_4_, 800 μL of 4% 2,2'-dipyridyl (in 70% *V*/*V* ethanol), and 400 μL of 3% (*m*/*V*) FeCl_3_ were added. The mixture was incubated at 42 °C for 45 min, and the absorbance at 525 nm was measured (model 8453; Agilent). Using the Lambert-Beer law, the AA concentration was obtained by reading the absorbance intensity. Results for PPhC were expressed as ChlA equivalents in mg per kg of fresh mass and for AA in mg per 100 g of fresh mass.

We selected these methods because they are widely used for vegetable matrices, and are validated by the Association of Official Analytical Chemists (AOAC) (*37*).

### Data proccesing and statical analysis

Data analyses were performed with OriginPro v. 2019^TM^ software (*38*). All experiments were performed using the Statgraphics Centurion XV v. 15.2.06 (*39*), and the results were represented as mean value±standard deviation (S.D.). The mean values of specific differences were analyzed by least significant difference (LSD) test, followed by the analysis of variance (ANOVA). The significance level of 0.05 was considered.

## RESULTS AND DISCUSSION

### Polyphenol detection in fresh-cut eggplant and carrot extracts

**Fig. 1:**
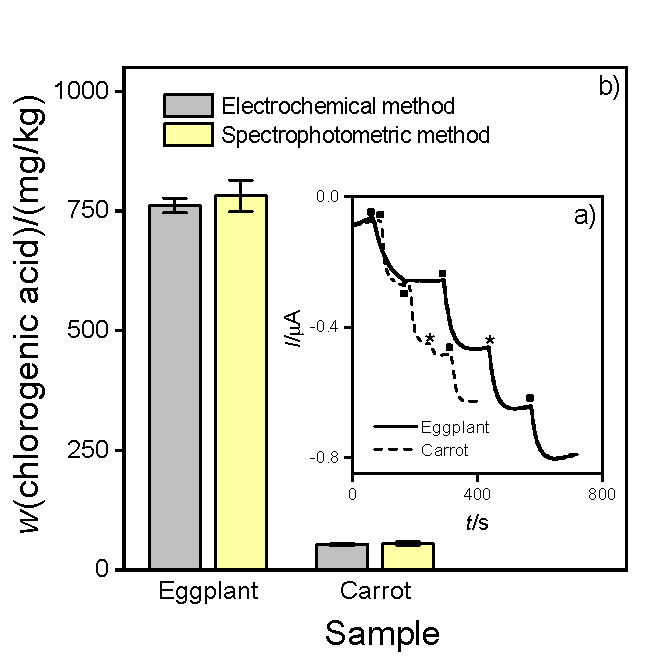
Images show: a) amperograms obtained with the electrochemical biosensor in 600 µM H_2_O_2_ and 0.1 M phosphate buffer (pH=5), after the addition of 0.85 µM chlorogenic acid (●) or sample (*): an aliquot of 10 μL fresh-cut eggplant (solid black line) and 20 μL carrot (dashed black line) extracts. Working potential: -200 mV, and b) polyphenolic compound content in fresh-cut eggplant and carrot extracts by electrochemical and spectrophotometric methods

The enzymatic biosensor was used to determine the PPhC content in fresh-cut eggplant and carrot extracts. Fig. 1a shows the bioelectrode current response profiles at working potential of -200 mV as a function of time in a cell containing 600 μM H_2_O_2_ in 0.1 M phosphate buffer at pH=5 (supporting electrolyte), after the addition of 0.85 μM ChlA standard solution and 10 and 20 μL fresh-cut eggplant and carrot samples prepared in triplicate, respectively, as indicated in the figure.

Before the quantification of the PPhC mass fraction in the fresh-cut eggplant and carrot extracts, the effects of the apparent resistivity (R_A_) on the possible interferences in the matrix were analyzed, which was calculated with the following equation (*36*):


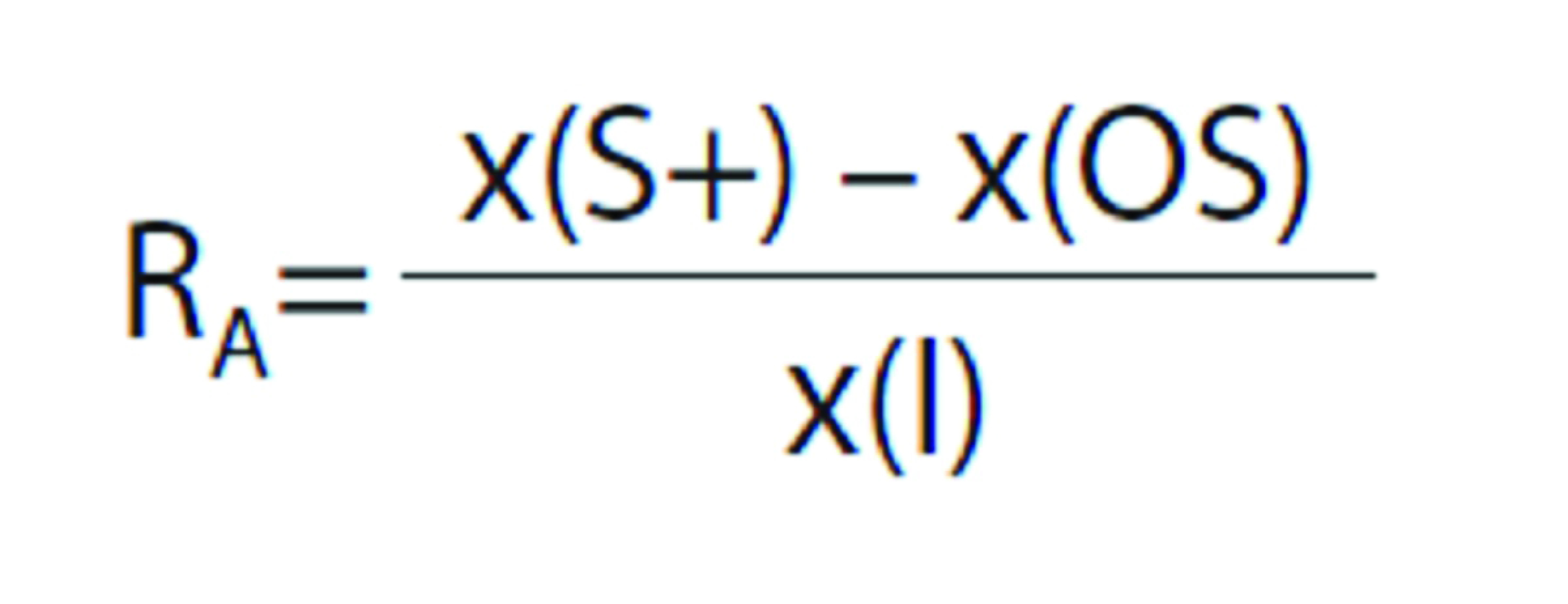


where x(S+I) is the measured response for the sample spiked with interference, x(S) is the measured value for the unspiked sample, and x(I) is the value of interference. Matrix effect is related to the possible interference of any component of the sample other than the analyte. The R_A_ expressed as percentage was 93 and 90% for eggplant and carrot extracts respectively. These R_A_ values indicate that any interference from any component of the sample (AA, for example) could be negligible.

Thereby, the electrochemical determination of the PPhC content was carried out in triplicate, obtaining the average values of (761±15) and (53±3) mg/kg for eggplant and carrot extracts, respectively.

To compare the results obtained with the electrochemical method, PPhC determination was also carried out with conventional spectrophotometric Folin-Ciocalteu method which measures the total antioxidant activity (*22*). Measurements were performed using 350 μL of the vegetable extracts, and absorbance was monitored in the 190-1100 nm range. The maximum absorbance at 765 nm allowed to determine the PPhC mass fraction (*N*=3) on fresh mass basis (782±33) and (55±4) mg/kg in eggplant and carrot extracts, respectively.

Fig. 1b shows the PPhC mass fraction obtained with electrochemical and spectrophotometric methods. Results showed that the determination of the PPhC content carried out by both methods was not significantly different, with a confidence level of 95%, even when working with different fruit and vegetable matrices. The convenience of the electrochemical biosensor to evaluate the PPhC content was demonstrated even when using a smaller sample volume.

### Ascorbic acid detection in eggplant and green leafy vegetable (rocket, lettuce and chard) extracts

**Fig. 2:**
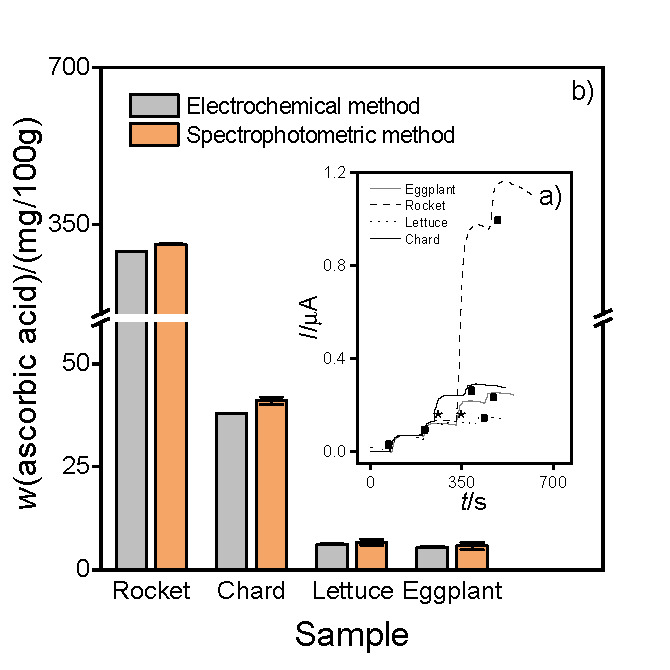
Images show: a) amperograms in 0.1 M phosphate buffer (pH=7) at 50 mV with the nanocomposite sensor after the addition of 1 µM ascorbic acid (●) or sample (*): an aliquot of 90 μL of fresh-cut eggplant (solid grey line) and 50 μL green leafy vegetables: rocket (dashed black line), lettuce (doted black line) and chard (solid black line) extracts. Working potential: 50 mV, and b) ascorbic acid content in fresh-cut eggplant and green leafy vegetables extracts by electrochemical and spectrophotometric methods

Fig. 2a shows the current time profiles for 1 µM AA standard solution and 90 and 50 µL eggplant and green leafy vegetable samples, respectively, prepared in triplicate. Measurements (*N*=3) were performed in 0.1 M phosphate buffer (pH=7) at 50 mV as working potential. The AA quantification was carried out in a similar way to that described for PPhC using the standard addition method, and evaluating the R_A_. The AA mass fraction on fresh mass basis was obtained as follows: (5.52±0.06), (290.00±0.06), (38.00±0.04) and (6.20±0.05) mg/100 g in eggplant, rocket, lettuce and chard, respectively. Furthermore, the apparent recoveries were 90% (eggplant), 92% (rocket), and 91% (lettuce and chard), indicating that any component in the matrix of these fruit and vegetables, other than the AA, does not significantly interfere with the signal.

The obtained values (Fig. 2b) were in agreement with those obtained with the spectrophotometric method described by Kampfenkel (*23*). AA spectrophotometric determination was carried out in triplicate; 200 μL fresh-cut eggplant and green leafy vegetable extracts were used and the absorbance intensity was monitored at 525 nm. The obtained values for AA mass fraction on fresh mass basis were: (5.8±0.9), (305±1), (41.0±0.9), and (6.7±0.7) mg/100 g for eggplant, rocket, lettuce and chard, respectively. As can be seen, there were no statistically significant differences between the results obtained by both methods, in a confidence interval of 95%. Therefore, the results showed the ability of the electrochemical sensor to determine the mass fraction of AA.

### Evaluation of fresh-cut eggplant exposed to UV-C radiation

To verify the versatility of the electrochemical method to detect small changes in the AA and PPhC contents, experiments similar to those described in the previous sections were performed. For this purpose, eggplants were previously irradiated with different UV-C light intensities, stored for different time periods, and prepared in triplicate according to the descriptions in *Extract preparation* (*40*).

#### Polyphenol detection

**Fig. 3:**
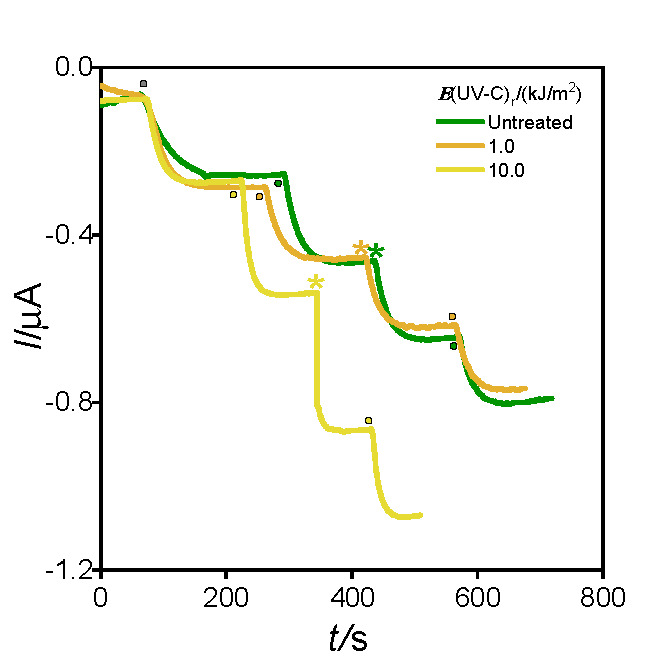
Amperograms obtained with the electrochemical biosensor in 600 µM H_2_O_2_ and 0.1 M phosphate buffer (pH=5) after the addition of 0.85 µM chlorogenic acid (●) or 10 μL of fresh-cut eggplant extract (*) (0 storage days) irradiated with different UV-C light intensities: untreated (green line), *E*_r_=1.0 orange line) and 10.0 kJ/m^2^ (yellow line). Working potential: -200 mV

Fig. 3 shows the corresponding amperograms obtained using the electrochemical biosensor (*N*=3) under the experimental conditions described in *Polyphenol detection in fresh-cut eggplant and carrot extracts* for the samples treated with different UV-C light intensities, on 0 storage days. >Insert here Fig. 3<

The average value of (687±10) mg/kg obtained for samples treated with the lowest UV-C light intensity (*E*_r_=1.0 kJ/m^2^) was similar to the control samples (761±9) mg/kg. However, samples irradiated with the highest intensity of UV-C light (*E*_r_=10.0 kJ/m^2^) showed an increase in the PPhC mass fraction (1030±9) mg/kg, as a protective response of the plant metabolism under stress. These results were in agreement with those obtained by the spectrophotometric method (*N*=3), (782±33), (692±33) and (1046±30) mg/kg for control samples and those treated with the lowest and highest UV-C intensity, respectively.

**Fig. 4:**
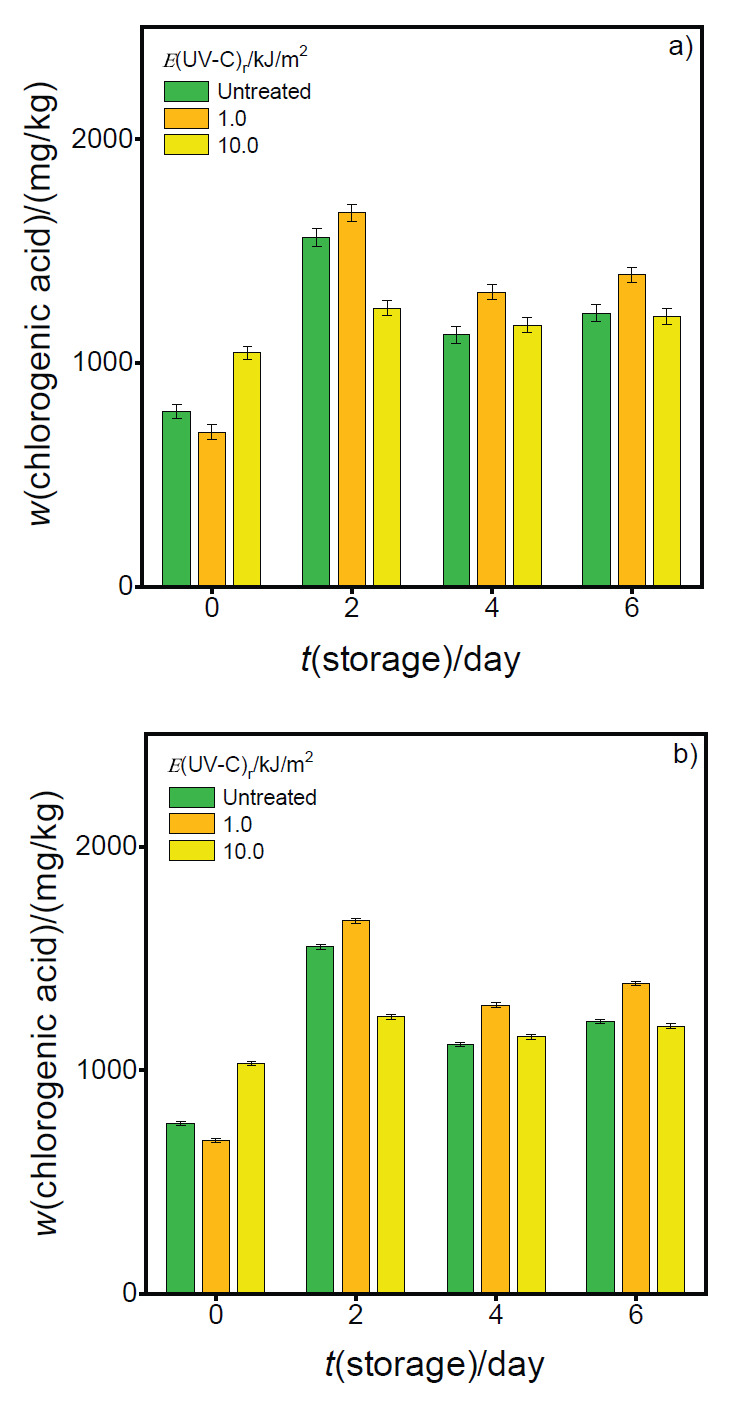
Polyphenolic compound mass fraction on fresh mass basis in fresh-cut eggplant extracts on 0, 2, 4 and 6 days of storage, irradiated with different UV-C light intensities: a) electrochemical and b) spectrophotometric methods

Fig. 4 gives the results of PPhC quantification by the electrochemical (Fig. 4a) and spectrophotometric (Fig. 4b) methods. The results indicated that samples not stored and treated with UV-C radiation intensity of 10.0 kJ/m^2^ showed a higher PPhC content than those irradiated with 1.0 kJ/m^2^ intensity or not irradiated at all. This could be related to a protective response of vegetable metabolism that has undergone great stress. Regardless of the applied radiation intensity, the change of the PPhC content during storage was similar. That is, it increased on the second day of storage, decreased on the fourth and did not significantly change on the sixth. Concellón *et al.* (*41*) and Barbagallo *et al.* (*42*) reported similar behaviour, observing an increase after refrigerated storage in whole and cut purple eggplants respectively, probably due to processing method and a greater bioavailability as a result of the activity of pectinase enzymes.

Our results demonstrate that the small variations that occur during storage can be satisfactorily detected by the proposed electrochemical method. Although Folin-Ciocalteu method has been widely used for this purpose, a thorough standardization of the method is required in terms of critical conditions such as proportion of reagents, temperature and reaction time (*43*). In this regard, the ability of the biosensor to monitor the changes in the PPhC content in fresh-cut eggplant exposed to UV-V treatments can be highlighted as a simple, fast, less-expensive, eco-friendly and low sample consumption method.

#### Ascorbic acid detection

**Fig. 5:**
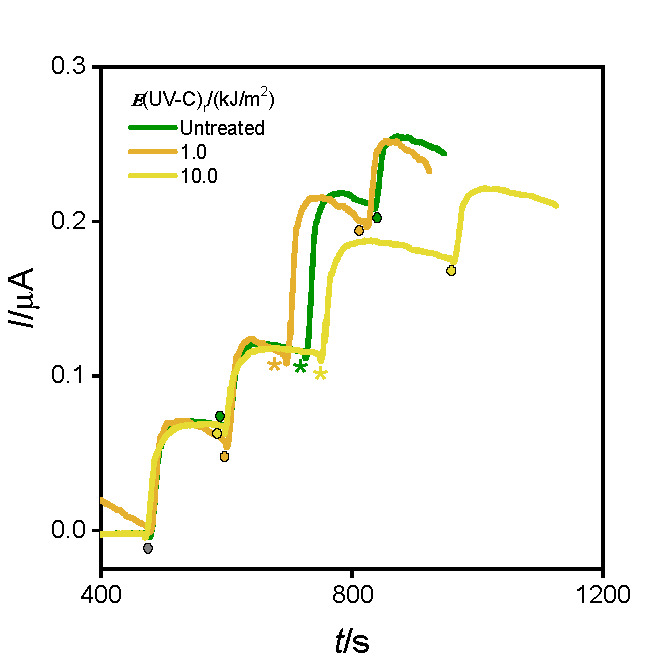
Amperograms obtained in 0.1 M phosphate buffer (pH=7) at 50 mV with the nanocomposite sensor after the addition of 1 µM AA (●) or 90 µL of fresh-cut eggplant extract (*) irradiated with different UV-C light intensities: untreated (green line), *E*_r_=1.0 (orange line) and 10.0 kJ/m^2^ (yellow line)

Fig. 5 shows the corresponding current profiles as a function of time obtained for each treatment on 0 storage days. The measurements were carried out in triplicate according to the descriptions in *Ascorbic acid detection in eggplant and green leafy vegetable (rocket, lettuce and chard) extracts.*

Results showed that AA mass fraction mean values on fresh mass basis in untreated samples ((5.52±0.06) mg/100 g) and in those exposed to lower UV-C light intensity ((5.47±0.06) mg/100 g) were similar to each other, but higher than the ones obtained for samples exposed to 10.0 kJ/m^2^ UV-C light intensity ((4.32±0.06) mg/100 g). On the other hand, the spectrophotometric method described by Kampfenkel (*23*) found (5.8±0.1), (5.8±0.3) and (4.8±0.1) mg/100 g (*N*=3) in the untreated sample and those irradiated with 1.0 and 10.0 kJ/m^2^, respectively, obtaining consistent results with both techniques.

**Fig. 6:**
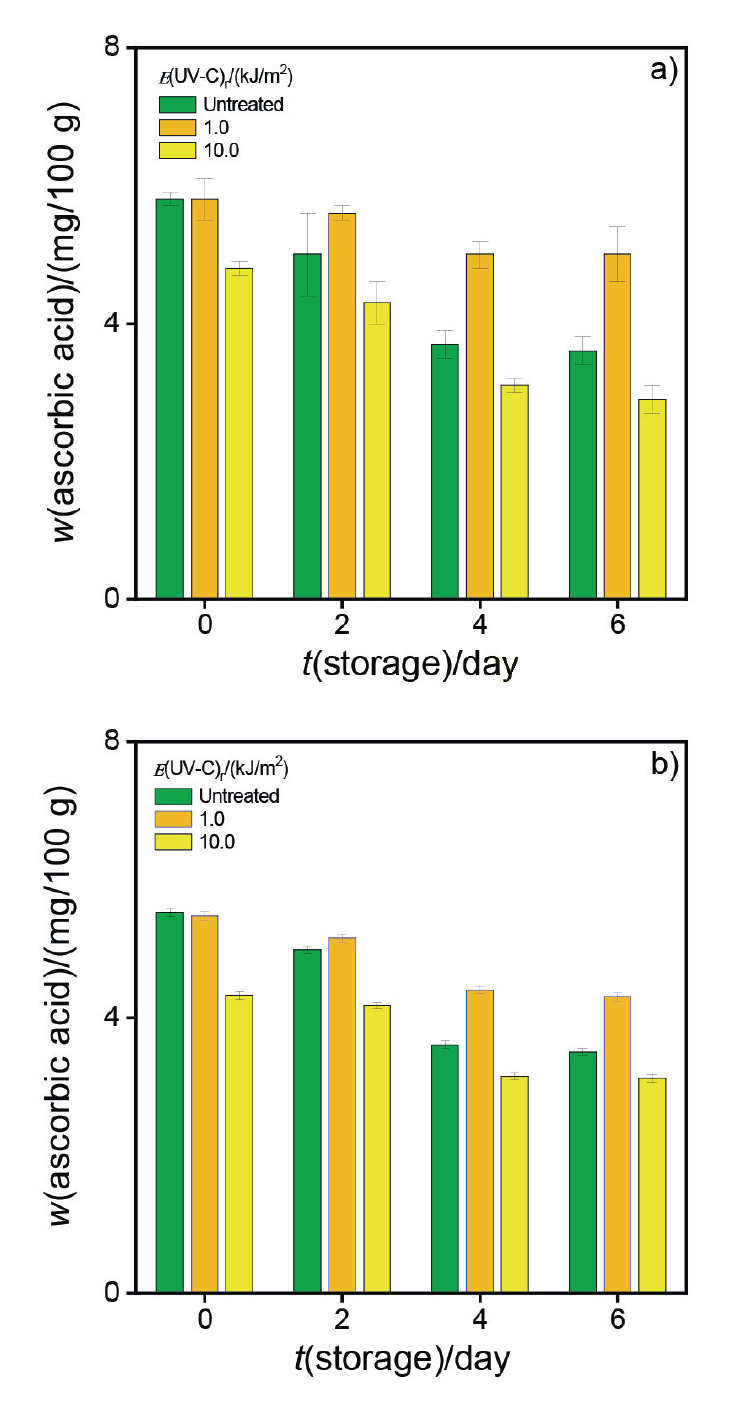
Ascorbic acid content in fresh-cut eggplant extracts on 0, 2, 4 and 6 days of storage, irradiated with different UV-C light intensities: a) electrochemical and b) spectrophotometric methods

Fig. 6 shows the AA mass fraction on 0, 2, 4 and 6 storage days achieved by electrochemical (Fig. 6a) and spectrophotometric (Fig. 6b) method.

The AA content decreased throughout storage. However, this decrease was more noticeable in the samples treated with radiation energy density 10.0 kJ/m^2^ than in those exposed to a lower UV-C light intensity or control samples. On the other hand, samples irradiated with 1.0 kJ/m^2^ UV-C light intensity had the highest AA mass fraction.

These results suggest that the metabolism and defence system of fresh-cut eggplant could be affected by the UV-C light intensity. Likewise, our results were consistent with those reported in the bibliography, indicating that UV-C radiation could be an appropriate tool to increase the content of different antioxidant compounds such as AA and, consequently, obtain fresh vegetables with desirable characteristics (*44*). Also, statistically significant differences between the results obtained by both methods are in a confidence interval of 95%.

Although the spectrophotometric and electrochemical methods show good analytical performance, the spectrophotometric one requires a longer analysis time and a more intensive sample preparation step to convert AA into a chromophoric product.

Therefore, the use of electrochemical sensors represents an attractive alternative to AA detection due to their remarkable characteristics such as short analysis time and small sample volume.

## CONCLUSIONS

The results obtained by electrochemical and spectrophotometric methods were in agreement. It was possible to determine the polyphenolic compounds and ascorbic acid in fresh-cut eggplants, carrots and green leafy vegetables (rocket, lettuce and chard). Likewise, the polyphenolic mass fractions obtained by both methods in fresh-cut eggplant exposed to different UV-C intensity radiation were similar, indicating the ability of the biosensor to monitor small variations. However, it is important to emphasize the advantages of using electrochemical sensors or biosensors such as higher selectivity and specificity, lower analysis and sample preparation time, smaller sample volume and less expensive equipment. Due to the advantages of the aforementioned electrochemical sensors, their application could not only be focused on the quantification of polyphenolic content and ascorbic acid in plant matrices, different foods, and samples of agri-food interest, but they could also monitor different stages in a production process.

## References

[1] Lobo V Patil A Phatak A Chandra N Free radicals, antioxidants and functional foods: Impact on human health. Pharmacogn Rev. 2010;4(8):118–26. 10.4103/0973-7847.70902 22228951 PMC3249911

[2] Kearney J Food consumption trends and drivers. Philos Trans R Soc Lond B Biol Sci. 2010;365(1554):2793–807. 10.1098/rstb.2010.0149 20713385 PMC2935122

[3] Bigliardi B Galati F Innovation trends in the food industry: The case of functional foods. Trends Food Sci Technol. 2013;31(2):118–29. 10.1016/j.tifs.2013.03.006

[4] Manzocco L Da Pieve S Bertolini A Bartolomeoli I Maifreni M Vianello A Surface decontamination of fresh-cut apple by UV-C light exposure: Effects on structure, colour and sensory properties. Postharvest Biol Technol. 2011;61(2–3):165–71. 10.1016/j.postharvbio.2011.03.003

[5] Roobab U Aadil RM Madni GM Bekhit AED The impact of nonthermal technologies on the microbiological quality of juices: A review. Compr Rev Food Sci Food Saf. 2018;17(2):437–57. 10.1111/1541-4337.12336 33350080

[6] Niakousari M, Gahruie HH, Razmjooei M, Roohinejad S, Greiner R. Effects of innovative processing technologies on microbial targets based on food categories: Comparing traditional and emerging technologies for food preservation. In: Barba FJ, Sant’Ana A, Orlien V, Koubaa M, editors. Innovative technologies for food preservation. Inactivation of spoilage and pathogenic microorganisms. London, UK: Elsevier Inc.; 2018. p. 133–85. https://doi.org/10.1016/B978-0-12-811031-7.00005-4

[7] McAuley CM Singh TK Haro-Maza JF Williams R Buckow R Microbiological and physicochemical stability of raw, pasteurised or pulsed electric field-treated milk. Innov Food Sci Emerg Technol. 2016;38(Part B):365–73. 10.1016/j.ifset.2016.09.030

[8] Zinoviadou KG Galanakis CM Brnčić M Grimi N Boussetta N Mota MJ Fruit juice sonication: Implications on food safety and physicochemical and nutritional properties. Food Res Int. 2015;77(4):743–52. 10.1016/j.foodres.2015.05.032

[9] Liao X Liu D Xiang Q Ahn J Chen S Ye X Inactivation mechanisms of non-thermal plasma on microbes: A review. Food Control. 2017;75:83–91. 10.1016/j.foodcont.2016.12.021

[10] Ramos-Villarroel AY Aron-Maftei N Martín-Belloso O Soliva-Fortuny R Influence of spectral distribution on bacterial inactivation and quality changes of fresh-cut watermelon treated with intense light pulses. Postharvest Biol Technol. 2012;69:32–9. 10.1016/j.postharvbio.2012.03.002

[11] Perkins-Veazie P Collins JK Howard L Blueberry fruit response to postharvest application of ultraviolet radiation. Postharvest Biol Technol. 2008;47(3):280–5. 10.1016/j.postharvbio.2007.08.002

[12] Mazurek S Fecka I Węglińska M Szostak R Quantification of active ingredients in *Potentilla tormentilla* by Raman and infrared spectroscopy. Talanta. 2018;189:308–14. 10.1016/j.talanta.2018.07.012 30086923

[13] Khodabakhshian R Feasibility of using Raman spectroscopy for detection of tannin changes in pomegranate fruits during maturity. Sci Hortic (Amsterdam). 2019;257:108670 10.1016/j.scienta.2019.108670

[14] Li X Sun C Luo L He Y Determination of tea polyphenols content by infrared spectroscopy coupled with iPLS and random frog techniques. Comput Electron Agric. 2015;112:28–35. 10.1016/j.compag.2015.01.005

[15] Islam MS Patras A Pokharel B Wu Y Vergne MJ Shade L UV-C irradiation as an alternative disinfection technique: Study of its effect on polyphenols and antioxidant activity of apple juice. Innov Food Sci Emerg Technol. 2016;34:344–51. 10.1016/j.ifset.2016.02.009

[16] Chen C Hu W He Y Jiang A Zhang R Effect of citric acid combined with UV-C on the quality of fresh-cut apples. Postharvest Biol Technol. 2016;111:126–31. 10.1016/j.postharvbio.2015.08.005

[17] Apak R Güçlü K Özyürek M Karademir SE Novel total antioxidant capacity index for dietary polyphenols and vitamins C and E, using their cupric ion reducing capability in the presence of neocuproine: CUPRAC method. J Agric Food Chem. 2004;52(26):7970–81. 10.1021/jf048741x 15612784

[18] Benzie IFF Starin JJ The ferric reducing ability of plasma (FRAP) as a measure of ‘“antioxidant power”’: The FRAP assay. Anal Biochem. 1996;239(1):70–6. 10.1006/abio.1996.0292 8660627

[19] Miller NJ Rice-Evans C Davies MJ Gopinathan V Milner A A novel method for measuring antioxidant capacity and its application to monitoring the antioxidant status in premature neonates. Clin Sci (Lond). 1993;84(4):407–12. 10.1042/cs0840407 8482045

[20] Brand-Williams W Cuvelier ME Berset C Use of a free radical method to evaluate antioxidant activity. Lebensm Wiss Technol. 1995;28(1):25–30. 10.1016/S0023-6438(95)80008-5

[21] Re R Pellegrini N Proteggente A Pannala A Yang M Rice-Evans C Antioxidant activity applying an improved ABTS radical cation decolorization assay. Free Radic Biol Med. 1999;26(9-10):1231–7. 10.1016/S0891-5849(98)00315-3 10381194

[22] Singleton VL Rossi JA Colorimetry of total phenolics with phosphomolybdic-phosphotungstic acid reagents. Am J Enol Vitic. 1965;16:144–58.

[23] Kampfenkel K Van Montagu M Inzé D Extraction and determination of ascorbate and dehydroascorbate from plant tissue. Anal Biochem. 1995;225(1):165–7. 10.1006/abio.1995.1127 10.1006/abio.1995.1127 7778771

[24] Khan MK Ahmad K Hassan S Imran M Ahmad N Xu C Effect of novel technologies on polyphenols during food processing. Innov Food Sci Emerg Technol. 2018;45:361–81. 10.1016/j.ifset.2017.12.006

[25] Grosjean R Le Godec Y Delacroix S Gouget G Beaunier P Ersen O A high pressure pathway toward boron-based nanostructured solids. Dalton Trans. 2018;47(23):7634–9. 10.1039/C8DT00932E 29796509

[26] Della Pelle F Compagnone D Nanomaterial-based sensing and biosensing of phenolic compounds and related antioxidant capacity in food. Sensors (Basel). 2018;18(2):462. 10.3390/s18020462 29401719 PMC5854963

[27] Della Pelle F Scroccarello A Sergi M Mascini M Del Carlo M Compagnone D Simple and rapid silver nanoparticles based antioxidant capacity assays: Reactivity study for phenolic compounds. Food Chem. 2018;256:342–9. 10.1016/j.foodchem.2018.02.141 29606458

[28] Vasilescu I Eremia SAV Kusko M Radoi A Vasile E Radu GL Molybdenum disulphide and graphene quantum dots as electrode modifiers for laccase biosensor. Biosens Bioelectron. 2016;75:232–7. 10.1016/j.bios.2015.08.051 26319166

[29] Vlamidis Y Gualandi I Tonelli D Amperometric biosensors based on reduced GO and MWCNTs composite for polyphenols detection in fruit juices. J Electroanal Chem (Lausanne). 2017;799:285–92. 10.1016/j.jelechem.2017.06.012

[30] Cerrato-Alvarez M Bernalte E Bernalte-García MJ Pinilla-Gil E Fast and direct amperometric analysis of polyphenols in beers using tyrosinase-modified screen-printed gold nanoparticles biosensors. Talanta. 2019;193:93–9. 10.1016/j.talanta.2018.09.093 30368304

[31] Tulli F Gulotta FA Martino DM Paz Zanini VI Borsarelli CD Ultrasensitive amperometric biosensing of polyphenols using horseradish peroxidase immobilized in a laponite/Au/DNA-bioinspired polycation nanocomposite. J Electrochem Soc. 2018;165(10):B452–7. 10.1149/2.1191810jes

[32] Tulli F Paz Zanini VI Fernández JM Martino DM López de Mishima BA Borsarelli CD Influence of electrostatic interactions induced *via* a nanocomposite film onto a glassy carbon electrode used for highly selective and sensitive ascorbic acid detection. J Electrochem Soc. 2019;166(8):B742–7. 10.1149/2.0011910jes

[33] Dahman Y Puskas JE Margaritis A Merali Z Cunningham M Novel thymine-functionalized polystyrenes for applications in biotechnology. Polymer synthesis and characterization. Macromolecules. 2003;36(7):2198–205. 10.1021/ma021608q

[34] Anastas PT, Warner JC. Green chemistry: Theory and practice. New York, NY, USA: Oxford University Press; 1998.

[35] Gutiérrez DR Char C Escalona VH Chaves AR Rodríguez SC Application of UV-C Radiation in the conservation of minimally processed rocket (*Eruca sativa* Mill.). J Food Process Preserv. 2015;39(6):3117–27. 10.1111/jfpp.12577

[36] Burns DT Danzer K Townshend A Use of the terms “recovery” and “apparent recovery” in analytical procedures (IUPAC recommendations 2002). Pure Appl Chem. 2002;74(11):2201–5. 10.1351/pac200274112201

[37] Kupina S Fields C Roman MC Brunelle SL Determination of total phenolic content using the Folin-C assay: Single-laboratory validation, First action 2017.13. J AOAC Int. 2018;101(5):1466–72. 10.5740/jaoacint.18-0031 29895350

[38] OriginPro, v. 2019^TM^, OriginLab Corporation, Northampton, MA, USA. 2019. Available from: https://www.originlab.com/index.aspx?go=COMPANY.

[39] Statgraphics Centurion XV. v. 15.2.06, Statgraphics Net, Madrid, Spain; 2007. Available from: https://statgraphics.net/.

[40] Lemos ML, Disalvo A, Rodríguez SC. Application of different doses of UV-C radiation to preserve the quality of IV range eggplants. Proceedings of the II Argentinian Congress of Biology and Postharvest Technology; 2019 September 11-13, Santiago del Estero, Argentina. pp. 107. (in Spanish).

[41] Concellón A Zaro MJ Chaves AR Vicente AR Changes in quality and phenolic antioxidants in dark purple American eggplant (*Solanum melongena* L. cv. Lucía) as affected by storage at 0 °C and 10 °C. Postharvest Biol Technol. 2012;66:35–41. 10.1016/j.postharvbio.2011.12.003

[42] Barbagallo RN Chisari M Caputa G Effects of calcium citrate and ascorbate as inhibitors of browning and softening in minimally processed ‘Birgah’ eggplants. Postharvest Biol Technol. 2012;73:107–14. 10.1016/j.postharvbio.2012.06.006

[43] Prior RL Wu X Schaich K Standardized methods for the determination of antioxidant capacity and phenolics in foods and dietary supplements. J Agric Food Chem. 2005;53(10):4290–302. 10.1021/jf0502698 15884874

[44] Santhirasegaram V Razali Z George DS Somasundram C Comparison of UV-C treatment and thermal pasteurization on quality of Chokanan mango (*Mangifera indica* L.) juice. Food Bioprod Process. 2015;94:313–21. 10.1016/j.fbp.2014.03.011

